# Malaria transmission pattern resilience to climatic variability is mediated by insecticide-treated nets

**DOI:** 10.1186/1475-2875-7-100

**Published:** 2008-06-02

**Authors:** Luis Fernando Chaves, Akira Kaneko, George Taleo, Mercedes Pascual, Mark L Wilson

**Affiliations:** 1 Department of Ecology and Evolutionary Biology, The University of Michigan, Ann Arbor, MI 48109-1048, USA; 2 Malaria Research, Unit of Infectious Diseases, Department of Medicine, Karolinska Institutet, 171 77 Stockholm, Sweden; 3 Department of International Affairs and Tropical Medicine, Tokyo Women's Medical University, Tokyo 162-8666, Japan; 4 Vanuatu Ministry of Health, Government of the Republic of Vanuatu, Port Vila, Vanuatu; 5 Department of Epidemiology, School of Public Health, The University of Michigan, Ann Arbor, MI 48109-2029, USA

## Abstract

**Background:**

Malaria is an important public-health problem in the archipelago of Vanuatu and climate has been hypothesized as important influence on transmission risk. Beginning in 1988, a major intervention using insecticide-treated bed nets (ITNs) was implemented in the country in an attempt to reduce *Plasmodium *transmission. To date, no study has addressed the impact of ITN intervention in Vanuatu, how it may have modified the burden of disease, and whether there were any changes in malaria incidence that might be related to climatic drivers.

**Methods and findings:**

Monthly time series (January 1983 through December 1999) of confirmed *Plasmodium falciparum *and *Plasmodium vivax *infections in the archipelago were analysed. During this 17 year period, malaria dynamics underwent a major regime shift around May 1991, following the introduction of bed nets as a control strategy in the country. By February of 1994 disease incidence from both parasites was reduced by at least 50%, when at most 20% of the population at risk was covered by ITNs. Seasonal cycles, as expected, were strongly correlated with temperature patterns, while inter-annual cycles were associated with changes in precipitation. Following the bed net intervention, the influence of environmental drivers of malaria dynamics was reduced by 30–80% for climatic forces, and 33–54% for other factors. A time lag of about five months was observed for the qualitative change ("regime shift") between the two parasites, the change occurring first for *P. falciparum*. The latter might be explained by interspecific interactions between the two parasites within the human hosts and their distinct biology, since *P. vivax *can relapse after a primary infection.

**Conclusion:**

The Vanuatu ITN programme represents an excellent example of implementing an infectious disease control programme. The distribution was undertaken to cover a large, local proportion (~80%) of people in villages where malaria was present. The successful coverage was possible because of the strategy for distribution of ITNs by prioritizing the free distribution to groups with restricted means for their acquisition, making the access to this resource equitable across the population. These results emphasize the need to implement infectious disease control programmes focusing on the most vulnerable populations.

## Background

Qualitative changes in the dynamics of populations, or regime shifts, are common phenomena across all living organisms [1,2]. Originally defined in fisheries science [3], the concept that at some time (termed a "breakpoint") there are disturbances that push a biological system beyond its normal dynamical pattern and can qualitatively change its behavior. Recently, this has become a major concern for vector-borne diseases in the context of global climatic change [4-6]. Such "breakpoints" derive from ecological analysis that has come to be known as Schmalhausen's law [2], which states that systems at the border of their limits of tolerance to one factor become more sensitive to small changes along any other dimension of existence [2]. Schmalhausen's law implies that if a system is pushed away from a state of exacerbation, its mean value and variability should decrease. This principle is strongly connected with the idea of resilience [7], the robustness of an ecological system before changing to a qualitatively different state, which in principle should be less susceptible to the effects of climatic variability as populations become less vulnerable to infection [8].

Malaria in the archipelago of Vanuatu has historically been a major public health problem as shown by the early entomological surveys of Buxton and Hopkins [9], followed by the extensive work of Bastien [10], where an increase in the burden of the disease in the early 1980s was reported [11], as well as its possible association to the evolution of quinine resistant parasites [12,13], numerous studies have shown this disease to be a major burden for Vanuatu inhabitants. Although occasionally hyperendemic, like in some areas of sub-Saharan Africa, malaria patterns are very different from this region in several aspects. In Vanuatu, the frequency of fatal cases is greatly diminished [14,15], the number of inapparent infections changes seasonally, disease depends on *Plasmodium *species [16], the diversity of parasites is reduced [17], and the genetic make-up of the native populations presents signatures of evolutionary changes driven by malaria. The latter is expressed in an increased frequency of α-thalassaemia associated with mild cases of malaria [18], and an increased frequency of G6PDH enzyme deficiency [19], which is different from sickle cell anaemia, the most common one seen in Africa [18,19].

Malaria control efforts also are important to analysis of this time pattern. In 1988, a major control intervention was launched, with a massive distribution of insecticide-treated nets (ITNs), following the abandon of indoor residual spraying for controlling malaria [20]. Although focused studies have demonstrated the use of ITNs to be very effective on small islands of this archipelago, as demonstrated by the elimination of the disease in Aneytium [21], another study analysing the effects of this policy at the level of the whole country has not been undertaken. In the present study, the dynamics of malaria before and after the introduction of ITNs into the archipelago are evaluated in an attempt to determine whether there were breakpoints where dynamics shifted transmission patterns, and quantified the effects of climate on these patterns before and after this intervention took effect.

## Methods

### Malaria data and monitored population at risk

**Figure 1:**
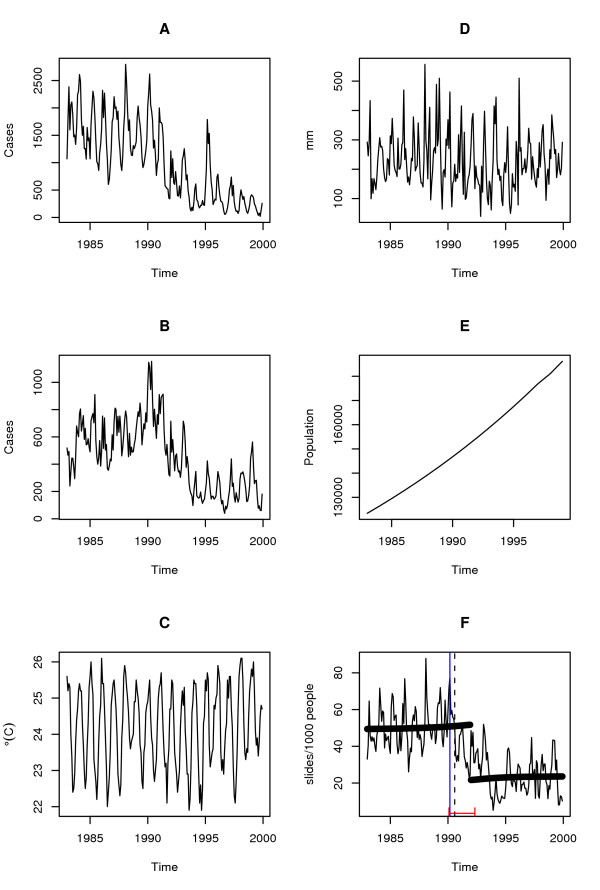
Time Series: **A ***Plasmodium falciparum *malaria cases, **B ***P. vivax *malaria cases, **C **Temperature (°C), **D **Precipitation (mm), **E **Population at risk (solid), **F **Monthly slide examination rate (slides*1000/population at risk), the dashed line corresponds to the breakpoint, August 1990, estimated using the F statistic, and the solid lines at the bottom of the graph to the confidence intervals (February 1990, May 1992) the thick-black solid line is the Kolmogorov-Zurbenko adaptive filter implemented with a half window size, q, of 36 months, the breakpoint is December 1991, the blue line corresponds to the breakpoint obtained using the CUSUM (march 1990). The mean rate (± S.D.) of slide examination before the breakpoint (August 1990) was (51.22 ± 11.40) being reduced to (25.92 ± 11.56) after it. Statistical tests of significance can be seen in Additional file 3.

**Figure 2:**
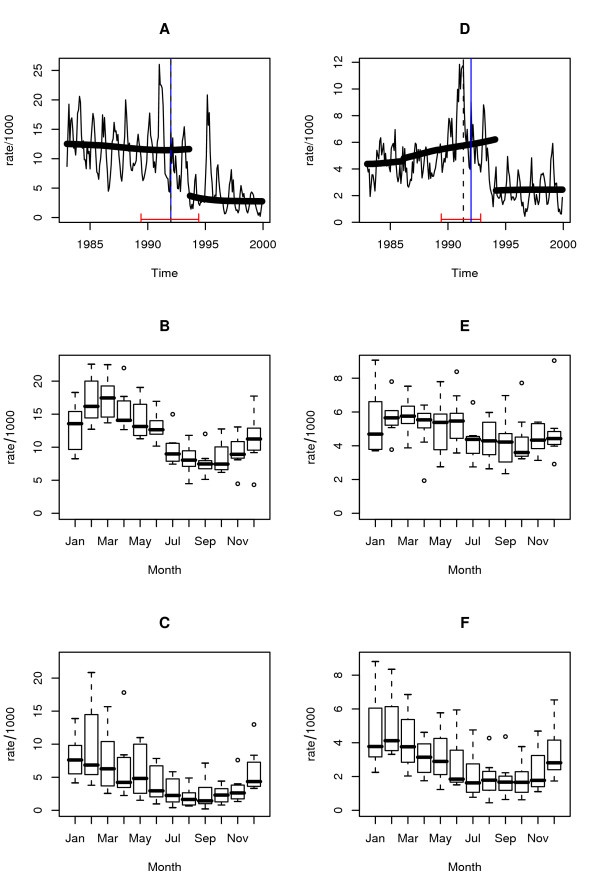
Regime Shift for falciparum and vivax malaria: **A **falciparum malaria rate, the dashed line corresponds to the breakpoint, January 1992, estimated using the F statistic, and the solid lines at the bottom of the graph to the confidence intervals (June 1989, June 1994), the thick-black solid line is the Kolmogorov-Zurbenko adaptive filter implemented with a half window size, q, of 36 months, the breakpoint corresponds to August 1993, the blue line corresponds to the breakpoint obtained using the CUSUM (January 1992).**B **&**C **seasonal falciparum malaria rate before and after breakpoint (January 1992) **D **vivax malaria rate, the dashed line corresponds to the breakpoint, May 1991, estimated using the F statistic, and the solid lines at the bottom of the graph to the confidence intervals (June 1989, November 1992), the black solid line is the Kolmogorov-Zurbenko adaptive filter implemented with a half window size, q, of 36 months, the breakpoint corresponds to February 1994, the blue line corresponds to the breakpoint obtained using the CUSUM (January 1992) **E **&**F **seasonal vivax malaria rate before and after breakpoint. For the F statistics the 30% percent of the data belonging to the extremes (15% each) was left out. For the Kolmogorov-Zurbenko adaptive filter q was set to 36, in order to avoid the misidentification of cycles shorter than 6 years.

**Figure 3:**
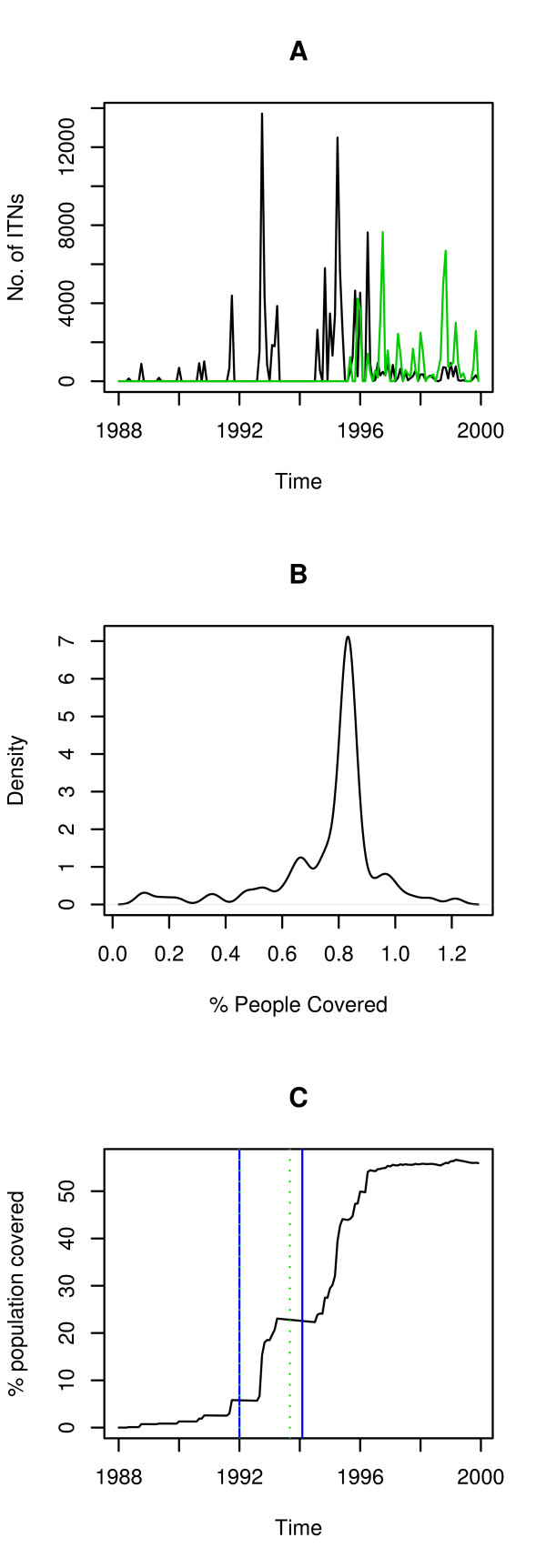
Bed nets **A **Monthly number of distributed bed nets(black line) and number Re-impregnated bed nets (green line) **B **Probability density of the percentage of people locally covered with bed nets between 1988 and 1997, bandwidth of 0.027 **C **Percent (%) of population covered by bed nets for the lower and upper time limit for the breakpoints, the green-blue line corresponds to January 1992 (*Plasmodium falciparum *and *P. vivax*), the green line to September 1992 (*P. falciparum*) and the blue line to December 1992 (*P. vivax*).

Monthly records of malaria were obtained from health centers of people who presented with fever or a recent history of fever, and whose standard blood slide analysis indicated infection with either *Plasmodium vivax *or *Plasmodium falciparum*, from January 1983 to December 1999. Malaria cases detected by this passive surveillance were the basis of the analysis. During this period total population increased (Figures 1, 2). Data on distributed ITNs with permethrin and re-impregnations were available for the same period (Figure 3A). Data collection was done under the guidance of the World Health Organization, and controlled by two of the authors (AK, GT) who maintained quality controls on the reporting system and diagnosis reliability during the studied period. All data were obtained from the Malaria and other Vector Borne Diseases Control Unit, Ministry of Health, Port Vila, Vanuatu.

This passive case detection system changed in January 1991, as slide examination in small rural health posts was discouraged by the central government of Vanuatu [19]. This policy change reduced the number of people being monitored, however, it remained representative of the whole population [19]. To account for the possible effects of this policy change, changes in the rate of slide examination were measured before and after the breakpoint obtained for the rate of slide examination, and assumed it to be linearly correlated to changes in the population monitored (Figure 1F). That is, the population at risk (corresponding to the population in districts where malaria was present) was multiplied by the fraction obtained by dividing the average rate of examination before and after the breakpoint to evaluate this possible source of error. A 50% reduction in average rate of slide examination was found during 1990–1991 (Figure 1F), as described in [19].

### Environmental data

Weather data included Sea Surface Temperature (SST) indexes: 1+2, 3, 3.4 and 4 (also known as the Niño 1+2, 3, 3.4 and 4 [22]; Additional file 1), and precipitation and temperature data from the climate database for political areas [23,24]. These data were used as predictors in models to assess changes in the magnitude of forcing by climatic variables in the dynamics of malaria incidence.

### Statistical analysis

#### Breakpoints and regime shifts

Tests of structural changes in time series can be undertaken using at least three different strategies: F tests that compare the null hypothesis of no regime shift to the presence of a regime shift, generalized fluctuation tests that do not assume any particular pattern of deviation from the absence of regime shifts [25,26] and adaptive filtering of signals [27]. A total of three approaches were used in the present study to assess the robustness of the findings. The F statistic is obtained by comparing the residuals $\hat{\varepsilon }$(*i*) of a segmented regression at time i with the residuals $\hat{\varepsilon }$ from an unsegmented regression using the following expression:

(1) $$F_{i}=\frac{{\hat{\varepsilon }}^{T}\hat{\varepsilon }-\hat{\varepsilon }{\left(i\right)}^{T}\hat{\varepsilon }(i)}{\hat{\varepsilon }{\left(i\right)}^{T}\hat{\varepsilon }(i)/(n-2k)}$$

Where n is the time series length and k the number of parameters. The null hypothesis is rejected when the supremum of the statistic is larger than the value of a distribution SupF derived by Hansen [28,29]. This approach has been generalized for *l *breaks, with arbitrary but fixed *l *[30,31]; where the number of breaks can be selected using conventional tools for model selection like the Akaike Information Criterion (AIC) [32].

The other two approaches, the generalized fluctuation test and the adaptive filtering, include formal significance tests, yet reveal regime shifts graphically instead of assuming specific types of departure in advance. For the generalized fluctuation test a parametric model is fitted to the data and an empirical process (EFP) is derived that captures the fluctuation either in residuals or parameter estimates [25,26]. Under the null hypothesis the fluctuations are governed by central limit theorems while under the alternative (regime shifts) the fluctuation is increased [26]. In the present analysis, the ordinary least squares (OLS) based CUSUM tests introduced in [33] was used. This test is based in cumulative sums of residuals from a linear regression:

(2) $$\begin{array}{cc} W_{n}^{0}(t)=\frac{1}{\hat{\sigma }\sqrt{n}}\sum_{i=1}^{\left\lfloor nt\right\rfloor }{\hat{\varepsilon }}_{i} & \left(0\leq \text{t}\leq 1\right) \end{array}$$

where a regime shift is evidenced by a single peak around the breakpoint, provided that the limiting process for $W_{n}^{0}(t)$ is the standard Brownian bridge *W*_0_(*t*) = *W*(*t*) - *tW*(l), where *W*(.) denotes Brownian motion. Significance for the CUSUM was tested using the derivations presented in [25,26]. For equation (1) and (2) the residuals $\hat{\varepsilon }$ came from a linear seasonal autoregressive [32] model fitted by OLS:

(3) *y*_*t *_= *μ *+ *φ*_1_*y*_*t*-1 _+ *φ*_12_*y*_*t*-12 _+ *ε*_*t*_

The third approach is totally non-parametric, and is based on recovering a signal and its breaks. The Kolmogorov-Zurbenko adaptive filter (KZAF) [27] is based on filtering the time series *y *using:

(4) $$z_{t}=\frac{1}{q_{H}(t)+q_{T}(t)}\sum_{i=-q_{T}(t)}^{q_{H}(t)}x_{t+i}$$

Where

(5) $$\begin{array}{l} q_{H}(t)=\{\begin{array}{ccc} q & \text{if} & D'(t)<0\\ f(D(t))q & \text{if} & D'(t)\geq 0 \end{array}\\ q_{T}(t)=\{\begin{array}{ccc} q & \text{if} & D'(t)>0\\ f(D(t))q & \text{if} & D'(t)\leq 0 \end{array} \end{array}$$

And q is half-length of a k iterative moving average (*x*_*t*_) applied to the original time series *y*_*t*_. The term *f*(*D*(*t*)) is defined by:

(6) $$f(D(t))=1-\frac{D(t)}{\max[D(t)]}$$

And D(t) is the absolute difference defined by:

(7) *D*(*t*) = |*x*_*t*+*q *_- *x*_*t*-*q*_|

And D'(t) as:

(8) *D*'(*t*) = *D*(*t *+ 1) - *D*(*t*)

Once *z*_*t *_is obtained quantitative estimates of discontinuity can be based on an analysis of the sample variances of *z*_*t*_, defined by:

(9) $${\hat{\sigma }}_{t}^{2}=\frac{\sum_{t=q_{T}}^{q_{H}}\left\{z_{t}-\bar{z}\right\}}{q_{T}+q_{H}}$$

When there are no breaks, maxima in the estimated variance of (9) are approximately independent and exponentially distributed with a expected number of peaks of about n/(2qk^0.5^), allowing to consider a breakpoint when the ${\hat{\sigma }}_{t}^{2}$ value exceeds the 95% upper tail of the exponential distribution with such parameter.

Regime shift analyses were carried out on: (i) the monthly rate of slide examination (No. Slides examined*1,000/Total population at risk); (ii) the monthly rate of the two malaria parasites (No. slides examined*1,000/Monitored population at risk) and (iii) weather variables (rainfall and temperature).

#### Threshold for ITN coverage

Time series for total number of bed nets distributed per month were accumulated and divided by the total population at risk estimated from the annual population data. It was assumed that the annual data corresponded to December, and interpolated the rest of the months using a smoothing splines regression as explained in [34]. The probability density [32] of the percentage of people locally covered with the distributed ITNs was also studied.

#### Seasonality

The seasonality of vivax and falciparum malaria rates (cases/population size) were assessed by using box diagrams before and after the regime shift [32].

#### Non-stationary patterns of association

The wavelet transform can be used to study the patterns of association between two nonstationary time series [35,36]. Specifically, the wavelet coherency analysis can determine whether the presence of a particular frequency at a given time in the disease corresponds to the presence of that same frequency at the same time in a covariate (e.g., rainfall and temperature). The cross-wavelet phase analysis can determine the time lag separating these two series as well.

#### Changes in the effects of climate on the dynamics

Once breakpoints for the regime shift were identified in the falciparum and vivax malaria rate series, the splitted series around the breakpoints were studied using seasonal auto-regressive (SAR) models [32]. The procedure for model building was similar to the one described in [36]: (i) a null model was fitted to the rate of the falciparum and vivax malaria (ii) temperature and rainfall were filtered with the coefficients of the null model, and (iii) cross-correlation functions were computed using the residuals of the null model and those of the filtered climatic variables.

The full model for *P. falciparum *considered precipitation (P) with lags of 2 and 29 months, and temperature (T) with lags of 3 and 12 months, as follows:

(10) *y*_*t *_= *μ *+ *φ*_1_(*y*_*t*-1 _- *μ*) + *φ*_12_(*y*_*t*-12 _- *μ*) + *β*_1_*P*_*t*-2 _+ *β*_2_*P*_*t*-29 _+ *α*_1_*T*_*t*-3_+ *α*_2_*T*_*t*-12 _+ *ε*_*t*_

For *P. vivax *the full model considered precipitation (P) a lag 9 months, and temperature (T) with a lag 10 months, as follows:

(11) *y*_*t *_= *μ *+ *φ*_1_(*y*_*t*-1 _- *μ*) + *φ*_12_(*y*_*t*-12 _- *μ*) + *β*_1_*P*_*t*-9 _+ *α*_1_*T*_*t*-10 _+ *ε*_*t*_

In both cases, the error was assumed as independent and normally distributed: *ε*_*t*_~*N*(0,$\sigma_{\varepsilon }^{2}$). After the initial fitting, models were simplified using a process of backward elimination[36]: (i) taking out one predictor at a time, (ii) finding the minimum AIC for models with similar complexity, i.e., number of parameters, (iii) comparing the likelihood of the best model (minimum AIC) for each level of complexity with the full model, and simplifying while differences were not statistically significant. For the analyses the climatic covariates were demeaned in order to not affect the intercept value [32].

## Results

Temporal patterns of malaria in Vanuatu present a clear shift in the incidence rate by the end of 1993 and beginning of 1994, for both parasite species (Figures 2A and 2D).

Breakpoints were confirmed by all three different methods (Additional files 2 and 3). For the incidence rate in both malaria species, breakpoints were statistically significant according to the F statistic and the variance of the KZAF. Even though the EFP estimates were not significant, peaks were detectable in both cases in January 1992 (Additional file 3). During that same time period no significant changes were found for climatic time series (Additional file 4). By the time changes were detected, bed net coverage (Figure 3B) was as low as 6% (EFP estimate) or slightly above 20% of the population at risk (KZAF). At a more local scale, villages where bed nets were distributed mostly had ~80% of the population covered (Figure 3C).

*Plasmodium falciparum *seasonality was qualitatively very similar before and after the breakpoint (Figures 2B and 2C), showing maximum incidence during the first quarter of the year (January-March), and minimum incidence during the third quarter of the year (July-September). For *P. vivax *(Figures 2E and 2F) a similar change was observed, although the patterns were not so clear as for *P. falciparum*, due to greater seasonal variability. With the exception of a brief period during 1992–1996 where cases due to both parasites were synchronous (i.e., with peaks at the same time), the dynamics of the infections were mainly asynchronous and not coherent (i.e., not associated in the frequency domain) at the seasonal scale. However, both diseases were significantly cross-coherent at an interannual scale, with the dynamics of *P. falciparum *cases being mostly synchronous with that of *P. vivax *(Additional file 5).

**Figure 4:**
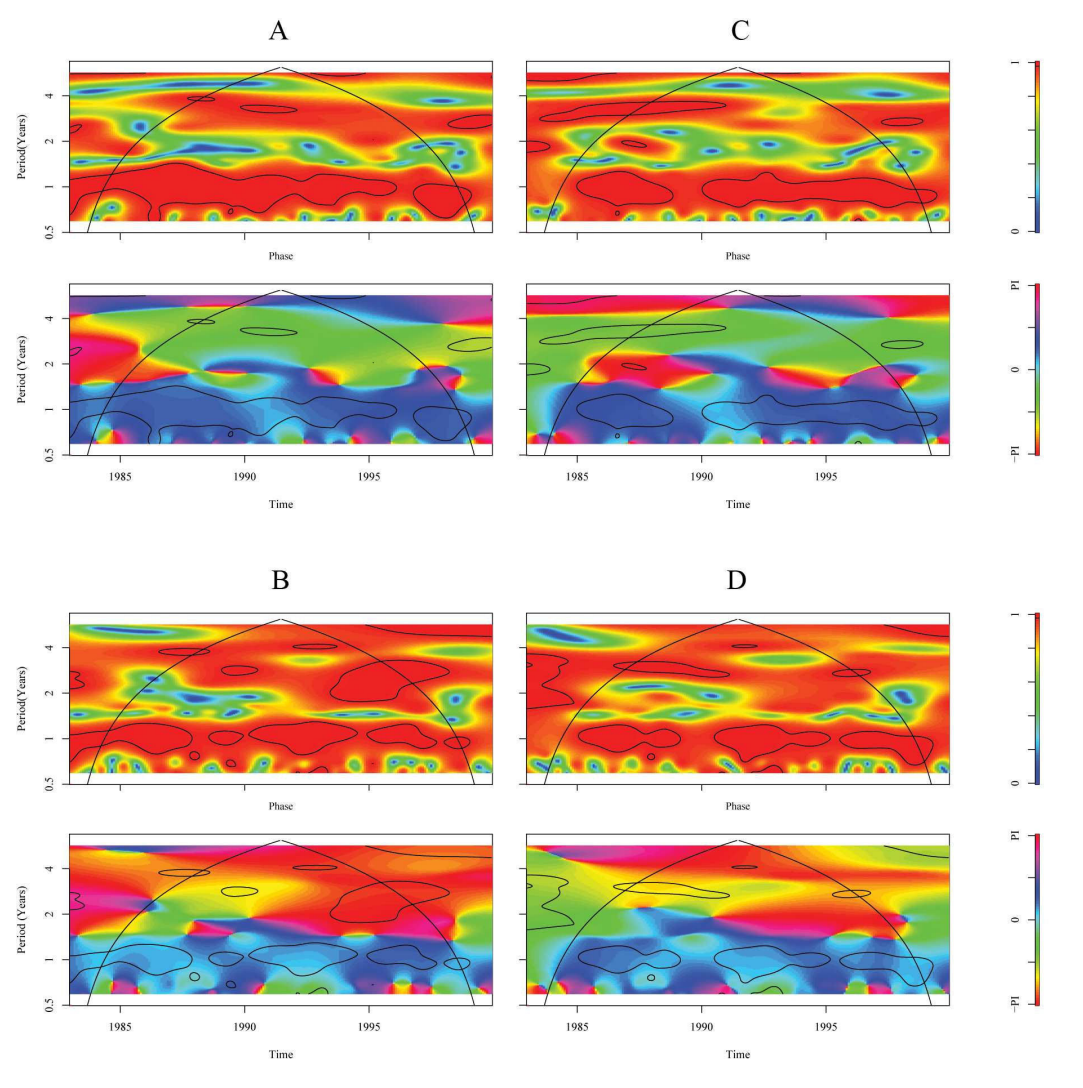
Cross-wavelet coherency and phase of *Plasmodium falciparum *malaria rate with **A **temperature and **B **rainfall and of *P. vivax *malaria rate with **C **temperature and **D **rainfall. The coherency scale is from zero (blue) to one (red). Red regions in the upper part of the plots indicate frequencies and times for which the two series share variability. The cone of influence (within which results are not influenced by the edges of the data) and the significant (*p *< 0.05) coherent time-frequency regions are indicated by solid lines. The colors in the phase plots correspond to different lags between the variability in the two series for a given time and frequency, measured in angles from -PI to PI. A value of PI corresponds to a lag of 17 mo. The procedures and software are those described in [31,32]. A smoothing window of 15 mo (2w + 1 = 31) was used to compute the cross-wavelet coherence.

Regarding the effects of climate during the studied period, the cross-coherence wavelet analysis showed malaria to be correlated with temperature at the seasonal scale; both *P. falciparum *and *P. vivax *incidence rates were led by temperature (Figure 4). A similar pattern was seen between the two parasites and rainfall at the seasonal scale, despite the presence of some gaps. A significant coherence with rainfall at interannual scales was also found. For *P. vivax*, coherence was statistically significant for periods between two and four years, during 1992–1996. No evidence that El Niño indices were leading the dynamics of the disease was identified.

Finally, Table 1 presents the parameter estimates for rate models of *P. falciparum *and *P. vivax*, including exogenous forcing by temperature, before and after the breakpoint. Model selection by backward elimination showed that rainfall was not a significant covariate (detailed values in Additional file 6). Following the qualitative change in the dynamics,*P. falciparum *had a proportional (~66%) and absolute (7.7 cases/1000 population) decline in incidence that was greater than that for *P. vivax *(~52% and 2.6 cases/1000 population, respectively). The importance of temperature in driving the dynamics also declined after the breakpoint for both species, between 31% and 49% for *P. falciparum *and 80% for *P. vivax *(the coefficient after the breakpoint became statistically non-significant). The importance of temperature in driving the dynamics also declined after the breakpoint for both species, between 31% and 49% for *P. falciparum *and, although not statistically significant, 80% for *P. vivax*. This suggests that the average effect of 1°C increase in temperature will increase incidence in a reduced amount when compared with its effect before the breakpoint. For example, preceding the shift each degree Celsius above the three-month lagged mean temperature value used to increase the rate by 1.43 cases/1000 people at risk, for *Plasmodium falciparum*. In contrast, after the breakpoint this change only increased the rate by half of its previous magnitude, i.e., 0.72/1000 people at risk (Table 1). A similar phenomenon was also seen for the variability that was not explained by the models, which also was reduced by 54% and 33% in *P. vivax *and *P. falciparum*, respectively, as shown by the decrease in the error variance of the models after the breakpoint (Table 1).

**Table 1:** Parameter values and % reduction for *Plasmodium falciparum *and *Plasmodium vivax *rate before and after the breakpoint obtained by using the variance of the Kolmogorov Zurbenko adaptive filter.

**Species**	**Parameter**	**Before**	**After**	**% Reduction**	**P**
***P.falciparum***	$\hat{\mu }$	11.56 ± 1.02	3.90 ± 1.15	66.26	B/A
	${\hat{\alpha }}_{1}$	1.43 ± 0.42	0.72 ± 0.35	48.59	B/A
	${\hat{\alpha }}_{2}$	1.36 ± 0.44	0.94 ± 0.33	30.88	B/A
	${\hat{\sigma }}_{\varepsilon }^{2}$	5.76	3.84	33.31	-

***P.vivax***	$\hat{\mu }$	4.83 ± 0.62	2.33 ± 0.43	51.76	B/A
	${\hat{\alpha }}_{1}$	0.55 ± 0.18	0.11 ± 0.16	80.00	B
	${\hat{\sigma }}_{\varepsilon }^{2}$	1.17	0.53	54.31	-

. % Reduction is defined as 1- (parameter value before breakpoint/parameter value after breakpoint). For *P. falciparum *the final model was:

*y*_*t *_= *μ *+ *φ*_1_(*y*_*t*-1 _- *μ*) + *φ*_12_(*y*_*t*-12 _- *μ*) + *α*_1_*T*_*t*-3 _+ *α*_2_*T*_*t*-12 _+ *ε*_*t *_and for *P.vivax*:

*y*_*t *_= *μ *+ *φ*_1_(*y*_*t*-1 _- *μ*) + *φ*_12_(*y*_*t*-12 _- *μ*) + *α*_1_*T*_*t*-10 _+ *ε*_*t *_Model selection process and all parameter values can be seen in Additional file 6. Column P (<0.05) indicates the significance of any parameter B (before breakpoint)/A(after breakpoint)

## Discussion

Following a disturbance, biological systems can either return to their normal state of variability or can move far away from such a state [1,2,37,38]. Transients, i.e., the anomalous behavior between regimes or basins [39,40], can obscure the qualitative changes of a system, because jumps from one state to another are not always instantaneous, complicating our ability to identify regime shifts [39,41]. This is likely one of the main differences between the dynamics of *P. falciparum *and *P. vivax*, since a consistent estimate for the breakpoint was easy to find for the former, while the estimates for the latter differed significantly. This was especially true for KZAF, which identified a later breakpoint. Assumptions underlying the employed techniques [25-33] might favour the estimate from KZAF, since the F statistic is quite sensitive to the stationarity (i.e., constant mean) of the time series, while the CUSUM EFP may be too sensitive given the quality of the data examined, identifying the change of policy in slide examination. By contrast, the KZAF is an adaptive technique that allows control of the time scale at which changes may be occurring [27]. This is a very useful characteristic for addressing one of the major recurrent problems in the study of ecological systems, i.e. finding the appropriate temporal scale of a natural phenomenon [42]. In this study, the adaptive ability of KZAF allowed for breaks to be distinguished from natural cycles associated with exogenous factors (i.e., climate). The fact that the basin (or regime) shift in the time series can be attributed to the effects of bed net use appears robust. During the study period no other major changes in control strategies, landscape cover, medication or drug resistance were reported [10,11,19] after controlling for the policy change in data collection [19].

The analysis identified a major difference between *P. falciparum *and *P. vivax*, namely the earlier breakpoint for *P. falciparum*. This pattern would not be expected under conditions of cross or heterologous immunity [43], and its evaluation with cross-infection studies is limited because quality data that are necessary to make such inferences [44] are lacking [19,21]. However, this pattern should be studied further, because it might reflect the dynamics of immunity in the population, where a generalized density-dependent immunity may be triggered by the within-host density of each parasite species [45]. Alternatively, if *P. falciparum *was the first species to be cleared, as shown in the classical co-infection neuro-syphilis malariotherapy experiments of Boyd and Kitchen [46], temporal patterns can only be appreciated when studying the dynamics of the within-host parasitic infection [47]. In addition, the pattern simply could arise by the ability of *P. vivax *to relapse [19,21], possibly in conjunction with the immunity dynamics described above.

Although regime shifts tend to be thought of in terms of increased variability as the best diagnostic condition [48], they can occur in the opposite direction, with systems becoming more stable. For both *P. falciparum *and *P. vivax *not only did the mean value of incidence decrease, but also the variance of the models decreased, which is a more robust measure of stability [38] than just looking at mean values [1] in dynamical systems. The patterns seen for the two species differed: falciparum malaria declined more abruptly, in total and relative terms, than in vivax malaria. Perhaps there are differences in the life history strategies of the parasites under different scenarios for transmission, with the most virulent parasite (*P. falciparum*) being more successful in environments with high transmission rates and the least virulent (*P. vivax*) being less sensitive to the intensity of transmission.

A surprising result was that the breakpoint occurred after just 20% of the population was covered with bed nets, which is half that predicted for *Anopheles gambiae *transmission by Killeen *et al *[49]. Perhaps *Anopheles farauti*, the main vector in Vanuatu [9,50] is less efficient. Regardless, the fact that such ITN coverage could explain the decrease has a robust theoretical explanation as presented in the groundbreaking work of Becker and Dietz [51], later confirmed using field data as the 80/20 rule for several infectious diseases [52,53] where the control, which targets 20% of the population, could benefit the other 80% of people.

Interestingly, this rule has been derived by looking at local populations, but the pattern seen in Vanuatu is more likely to arise from the subdivided nature of the population in villages, or patches if seen from the perspective of metapopulations [54]. The coverage per patch was high enough (80% with a very low dispersion around this value) to guarantee the local interruption of transmission according to mechanistic models of bed net action in settings with a higher entomological inoculation rate [49,55] than that observed in Vanuatu [16,50].

As a control strategy, ITNs outperform similar strategies aimed at reducing vectorial capacity, such as the indoor residual spraying, mainly because of its cost-effectiveness, as well as for its ease of implementation and distribution [56,57]. Several studies have shown that bed nets reduce total infant mortality in endemic areas [58,59], are a sustainable option for control in terms of the reduction of relative risk of malaria death in the medium- to long-term time scales [60], and are successful across several cultural settings [57,61-64]. The advantages of bed nets also go beyond the immediate effects, since so far there is no evidence for selection of insecticide-resistant mosquitoes [65], and they are protective even in areas where mosquito resistance to the insecticides used for bed net impregnation has been reported [66]. This result also has been theoretically reinforced by models that consider the use of bed nets in conjunction with other control strategies, such as zooprophylaxis [67], provided that both measures in conjunction are likely to counteract any selective pressure for the development of insecticide resistance, since mosquito fitness would not be under a selective pressure, and may even be under selection for feeding preferences in non-human hosts [68,69]. However, urban settings pose a major challenge since effective zooprophylaxis might be diminished because of higher human densities. Behavioral changes in mosquitoes and decreased bed net effectiveness have been documented in urban areas [70]. From a wider perspective, bed nets are also a more ecologically-sound strategy since they reduce impacts on natural enemies of vectors via positive feedbacks loops that can be generated by large scale insecticide spraying [68,71,72]. A large body of literature supports that idea that in relatively undisturbed environments mosquito abundance is regulated by interactions with other animals, e.g., tadpoles, fish and other insects [e.g., [71-76]], however such natural control is diminished by anthropogenic disturbances of food webs.

## Conclusion

The success of the Vanuatu malaria control programme also stems from the strategy of bed net distribution, where large fractions of the population were locally covered at the village level, ensuring the reduction in transmission, even leading to local elimination in some islands [21]. As stressed by Killeen *et a*l [49] and Ilboudo-Sanogo *et al *[65], an efficient bed net programme needs to cover a large proportion of the population in order to ensure that both sources (e.g., asymptomatic people) and sinks (e.g., pregnant women and young children) of infection are effectively covered. The erroneous targeting of transmission groups for control can exacerbate the conditions for transmission [77]. Additionally, as suggested by Mathanga *et al *[78], for ethical and humanitarian reasons the goal should be to cover as much of the population present in the endemic setting as possible, retaining traditional practices (e.g., voluntary work) for the exchange of goods when mainstream means of commercialization are not enough to achieve such a goal. In Vanuatu, special care was taken to address these factors by implementing a strategy where children under five years of age, their mothers and pregnant women received free nets. Cost was half price for school children and other adults were charged the full price, ensuring an equitable coverage of the population [21] and an equitable distribution of this valued resource.

A factor that deserves further study is the role that concomitant knowledge transfer associated to the distribution of bed nets have on the awareness of the population about the risk leading to malaria transmission. Unlike insecticide residual spraying whose effectiveness depends mostly on being applied correctly, the effective use of bed nets requires knowledge for its proper use. In Vanuatu, parents' awareness was likely to play a role in diminishing incidence among young children (<5 years), because of the free distribution to this age group and training to parents about the benefits of using the nets [21]. But, the positive effects of knowledge transfer are likely to be more comprehensive. For example, Mathanga *et al *[78] showed that even though children didn't regularly use bed nets, those in communities where malaria transmission plummeted after the introduction of widespread bed net use were aware of the benefits. Similar knowledge transfers are known to be present among some Native American tribes whose mythology has associated malaria risk with the blossoming of water-retaining flowers where vector larvae develop [79]. Changes in collective behavior in villages that were stricken by malaria have been seen before community-based educational campaigns were implemented [80-82] and more generally, traditional knowledge has been shown to be a robust strategy to handle issues of pest management by native populations in Meso-America [83].

The association between climatic forces and malaria dynamics in Vanuatu presents features that make it unusual when compared to other settings, where the climate and ecological dynamics have been studied [e.g., [36], reviewed in [84]]. None of the ENSO indices led the dynamics of malaria, yet clear signals of association at interannual time scales were found with local climatic variables. This may be a result of the relationship of ENSO with the local climate in the area [85] which influences rainfall during a season, October to January [86-88], that probably is not relevant for the biology of mosquitoes in regards to transmission. The unusual pattern is less likely because of a demographic effect of small insular population size as suggested in [89]. Mechanisms for the action of rainfall across a wide range of landscapes have been very well described, it increases the rate of a disease when new mosquito habitats are created by increased precipitation [90], and the additional weakening of inter-specific interactions regulating mosquito populations [91]. However, ecological studies of vectors are needed to understand their local population dynamics in Vanuatu. Similarly it may be understood why hotter temperatures can increase the transmission of vector-borne diseases, because of known effects of temperature on the rate of insect and parasite development [85,92]. However, increased resilience to the effects of climate in an infectious disease as a result of control measures, in our knowledge, has not been reported before. The fact that such a measure also decreases the incidence of malaria under changing climatic conditions is a remarkable fact strengthening the usefulness of this strategy.

Finally, a precautionary note on bed nets should be posed. Even though they are a very robust strategy to control malaria from evolutionary, ecological, conservation and cost-effectiveness perspectives [56,57,65,78], the use of bed nets should not be viewed as a exhaustive solution if the long-term goal of population health is to be pursued. As shown in [93] a fraction of the death toll that was avoided by controlling malaria through the use of insecticide treated curtains in areas of Burkina Faso was shifted to meningococcal meningitis. Evidence also suggests that in urban settings, for a series of factors that go from the absence of alternative hosts to behavioural shifts in humans, insecticide treated nets are not going to be a sufficient strategy to keep malaria under control [70]. To achieve this goal, a wide research agenda, fully integrated with policies beyond disease control is a path that needs to be taken [34,94-97], where ultimate goals are aimed at pushing out the stressful contextual conditions that make human populations vulnerable to infectious diseases [2], especially malaria.

## Competing interests

The authors declare that they have no competing interests.

## Authors' contributions

LFC conceived the study and carried out the analyses. AK and GT collected the data and performed the field studies. AK and GT provided input on methods. AK, MP, MLW provided input on results interpretation. LFC drafted the manuscript to which AK, GT, MP, and MLW made contributions.

## Supplementary Material

Additional file 1 Time Series for The El Niño Southern Oscillation: **A **SST 1+2, **B **SST 3, **C **SST 3.4, **D **SST 4. Click here for file

Additional file 2 Breakpoints for the rate of slide examination **A **F statistic for the falciparum malaria rate, the solid line is the 95% upper tail of the distribution for the F Statistic [24,25]**B **Empirical fluctuation period of the CUSUM test, the maximum value is in may 1992, the redline is the threshold value for breakpoint significance [23,24]**C **Variance of the Kolmogorov-Zurbenko adaptive filter, the dashed line is the 95% upper tail of the exponential distribution for this statistic, the maximum value corresponds to may 1992. Click here for file

Additional file 3 Breakpoints for the *Plasmodium falciparum *and *P. vivax *rates **A **F statistic for the falciparum malaria rate, the solid line is the 95% upper tail of the distribution for the F Statistic [24,25]**B **Empirical fluctuation period of the CUSUM test, the maximum value is in may 1992 **C **Variance of the Kolmogorov-Zurbenko adaptive filter, the dashed line is the 95% upper tail of the exponential distribution for this statistic, the maximum value corresponds to may 1992 **D **F statistic for the vivax malaria rate, the solid line is the 95% upper tail of the distribution for the F Statistic [24,25]**E **Empirical fluctuation period of the CUSUM test for, the maximum value is in January 1992 **F **Variance of the Kolmogorov-Zurbenko adaptive filter. In **A**, **B **and **D, E **the redline is the threshold value for breakpoint significance [23,24]. In **C **and **F **the blue dashed line is the 95% upper tail of the exponential distribution for this statistic, the maximum value corresponds to December 1992. Click here for file

Additional file 4 Breakpoints for **A,B,C **Temperature and **D,E,F **Rainfall in Vanuatu using the F statistics(**A,D**), the empirical fluctuation period of the CUSUM (**B,E**) and the Kolmogorov-Zurbenko Adaptive Filter, KZAF (**C,F**). For the F statistics the 30% percent of the data belonging to the extremes was left out. For the KZAF only the seasonality was filtered (i.e., q = 6) Click here for file

Additional file 5 Cross-wavelet coherency and phase of *Plasmodium vivax *malaria rate with *P. falciparum *malaria rate. For technical details see legend of Figure 4. Click here for file

Additional file 6 Model selection and parameter values for models of *Plasmodium falciparum *and *Plasmodium vivax *rates before and after the breakpoint found using the Kolgomorov Zurbenko Adaptive filter. Click here for file
