# Supplementary material for: Malaria transmission pattern resilience to climatic variability is mediated by insecticide-treated nets
Source: Malar J. 2008 Jun 2;7:100. doi: 10.1186/1475-2875-7-100 (PMC2443810; doi:10.1186/1475-2875-7-100)
Supplement: Additional file 6 — Model selection and parameter values for models of Plasmodium falciparum and Plasmodium vivax rates before and after the breakpoint found using the Kolgomorov Zurbenko Adaptive filter. [file 1475-2875-7-100-S6.doc]

**Additional file 6**

| Parasite/  Breakpoint | | $\hat{\varphi}_{1}$ | $\hat{\varphi}_{12}$ | $\hat{\alpha}_{1}$ | $\hat{\alpha}_{2}$ | $\hat{\beta}_{1}$ | $\hat{\beta}_{2}$ | $\hat{\mu}$ | $\hat{\sigma}_{\varepsilon}$ | $p_{\chi^{2}}$ | d.f. | Akaike Information Criterion |
| --- | --- | --- | --- | --- | --- | --- | --- | --- | --- | --- | --- | --- |
| *Plasmodium falciparum* | Before Breakpoint | 0.72 ± 0.06 | 0.36 ± 0.10 | 1.38 ± 0.42 | 1.44 ± 0.46 | 0.10 ± 0.51 | 0.83 ± 0.57 | 11.51 ± 1.09 | 2.38 | — | — | 603.32 |
|  |  | 0.70 ± 0.07 | 0.34 ± 0.10 | 1.41 ± 0.42 | 1.35 ± 0.44 | 0.13 ± 0.52 | — | 11.56 ± 1.02 | 2.40 | 0.14 | 1 | 603.42 |
|  |  | 0.72 ± 0.06 | 0.36 ± 0.10 | 1.39 ± 0.42 | 1.45 ± 0.45 | — | 0.84 ± 0.57 | 11.51 ± 1.08 | 2.37 | 0.83 | 1 | 601.37 |
|  |  | 0.74 ± 0.06 | 0.48 ± 0.08 | 1.28 ± 0.46 | — | 0.39 ± 0.61 | 0.26 ± 0.51 | 11.34 ± 1.48 | 2.44 | <0.001 | 1 | 610.09 |
|  |  | 0.74 ± 0.06 | 0.43 ± 0.09 | — | 1.28 ± 0.49 | 0.28 ± 0.52 | 0.87 ± 0.57 | 11.34 ± 1.37 | 2.46 | <0.005 | 1 | 611.18 |
|  |  | 0.70 ± 0.07 | 0.33 ± 0.10 | 1.43 ± 0.42 | 1.36 ± 0.44 | — | — | 11.56 ± 1.02 | 2.40 | 0.33 | 2 | 601.48 |
|  |  | 0.74 ± 0.06 | 0.48 ± 0.08 | 1.30 ± 0.46 | — | — | 0.68 ± 0.57 | 11.34 ± 1.47 | 2.45 | <0.005 | 2 | 608.35 |
|  |  | 0.74 ± 0.06 | 0.43± 0.09 | — | 1.30 ± 0.48 | — | 0.88 ± 0.57 | 11.34 ± 1.37 | 2.46 | <0.01 | 2 | 609.48 |
|  |  |  |  |  |  |  |  |  |  |  |  |  |
|  |  |  |  |  |  |  |  |  |  |  |  |  |
|  | After Breakpoint | 0.84 ± 0.06 | -0.01 ± 0.12 | 0.65 ± 0.33 | 1.11 ± 0.31 | 0.70 ± 0.55 | 0.89 ± 0.42 | 4.07 ± 1.26 | 1.89 | — | — | 329.76 |
|  |  | 0.82 ± 0.06 | -0.01 ± 0.12 | 0.63 ± 0.35 | 0.97 ± 0.32 | 0.63 ± 0.57 | — | 3.98 ± 1.15 | 1.94 | <0.05 | 1 | 332.00 |
|  |  | 0.83 ± 0.06 | 0.04 ± 0.12 | 0.75 ± 0.33 | 1.07 ± 0.33 | — | 0.86 ± 0.43 | 3.98 ± 1.25 | 1.91 | 0.75 | 1 | 329.35 |
|  |  | 0.82 ± 0.02 | 0.15 ± 0.12 | 0.52 ± 0.39 | — | 0.39 ± 0.59 | 0.68 ± 0.46 | 3.91 ± 1.39 | 2.02 | <0.005 | 1 | 337.76 |
|  |  | 0.84 ± 0.06 | 0.05 ± 0.12 | — | 1.05 ± 0.33 | 0.90 ± 0.56 | 0.86 ± 0.43 | 4.01 ± 1.37 | 1.93 | 0.06 | 1 | 331.27 |
|  |  | 0.81 ± 0.06 | 0.04 ± 0.12 | 0.72 ± 0.35 | 0.94 ± 0.33 | — | — | 3.90 ± 1.15 | 1.96 | 0.07 | 2 | 331.19 |
|  |  | 0.82 ± 0.06 | 0.17± 0.11 | 0.57 ± 0.39 | — | — | 0.68 ± 0.46 | 3.87 ± 1.41 | 2.02 | <0.05 | 2 | 366.24 |
|  |  | 0.83 ± 0.06 | 0.14 ± 0.11 | — | 0.96 ± 0.35 | — | 0.81 ± 0.45 | 3.87 ± 1.43 | 1.96 | 0.11 | 2 | 366.91 |
|  |  |  |  |  |  |  |  |  |  |  |  |  |
|  |  |  |  |  |  |  |  |  |  |  |  |  |
|  | |  |  |  |  |  |  |  |  |  |  |  |
| *Plasmodium. vivax* | Before Break-point | 0.81 ± 0.05 | 0.26 ± 0.09 | 0.47 ± 0.18 | — | -0.38 ± 0.22 | — | 4.88 ± 0.61 | 1.07 | — | — | 411.31 |
|  |  | 0.80 ± 0.05 | 0.27 ± 0.08 | 0.55 ± 0.18 | — | — | — | 4.83 ± 0.62 | 1.08 | 0.09 | 1 | 412.18 |
|  |  | 0.81 ± 0.05 | 0.31 ± 0.09 | — | — | -0.51 ± 0.22 | — | 4.83 ± 0.66 | 1.09 | <0.01 | 1 | 415.94 |
|  | After Break-point | 0.71 ± 0.09 | 0.36 ± 0.15 | 0.11 ± 0.17 | — | -0.13 ± 0.18 | — | 2.33 ± 0.42 | 0.73 | — | — | 166.98 |
|  |  | 0.72 ± 0.08 | 0.36 ± 0.13 | 0.11 ± 0.16 | — | — | — | 2.33 ± 0.43 | 0.73 | <0.02 | 1 | 166.75 |
|  |  | 0.71 ± 0.09 | 0.40 ± 0.13 | — | — | -0.14 ± 0.18 | — | 2.32 ± 0.45 | 0.73 | <0.02 | 1 | 165.35 |

The parameters for the full model are in the first data row, and for the null model are in the last data row. Parameters are described in the text and are given as value ± standard error. $p_{\chi^{2}}$ is the significance of the chi-squared likelihood ratio test between each model and the full model, and d.f. its degrees of freedom.
